# The influence of parents on children’s consciousness of gender equality: a multi-group structural equation modeling approach

**DOI:** 10.3389/fpsyg.2024.1361281

**Published:** 2024-08-14

**Authors:** Yifei Li, Jie Zhang, Juan Li, Yiping Chen, Jingping Zhang, Man Zuo

**Affiliations:** ^1^Xiangya Nursing School of Central South University, Changsha, Hunan, China; ^2^Hunan University of Chinese Medicine, Changsha, Hunan, China; ^3^Department of Gastroenterology, Shenzhen Hospital, Southern Medical University, Shenzhen, Guangdong, China; ^4^Heyuan People’s Hospital, Heyuan, Guangdong, China

**Keywords:** school-age children, consciousness of gender equality, ecological systems theory, structural equation modeling, parents, gender role

## Abstract

**Objective:**

This study aimed to understand the consciousness of gender equality among school-aged children in China and its influencing factors using structural equation modeling to explore the pathways, intensity and group differences among these factors.

**Methods:**

A cross-sectional survey was conducted using stratified random whole-group sampling of primary school students in grades 1–6 and their parents who met the inclusion and exclusion criteria. In this study, 1,312 valid questionnaires were collected from a total of 1,500 school-aged children in Hunan Province, China (effective response rate of 87.5%). Statistical analysis was conducted using SPSS 26.0 and AMOS 24.0 software. Statistical inference consisted of *t*-tests, analysis of variance, the LSD test, Pearson correlation analysis, multiple stepwise linear regression analysis and structural equation modeling.

**Results:**

School-aged children had the lowest consciousness of gender equality in the area of occupation and relatively higher consciousness in the areas of family and school. Children’s age, gender, gender role, parent–child relationship, teacher-student relationship and parents’ gender equality consciousness had predictive effects on children’s consciousness of gender equality. The structural equation model constructed in this study is applicable to school-aged children of different genders. There was a significant difference in the structural equation modeling for children in different study period groups.

**Conclusion:**

In the education process, parents and teachers should attempt to improve their own consciousness of gender equality, integrate the concept of androgynous education, enhance close relationships with children, and adopt appropriate education methods according to the characteristics of different groups of children.

## 1 Introduction

**Figure 1:**
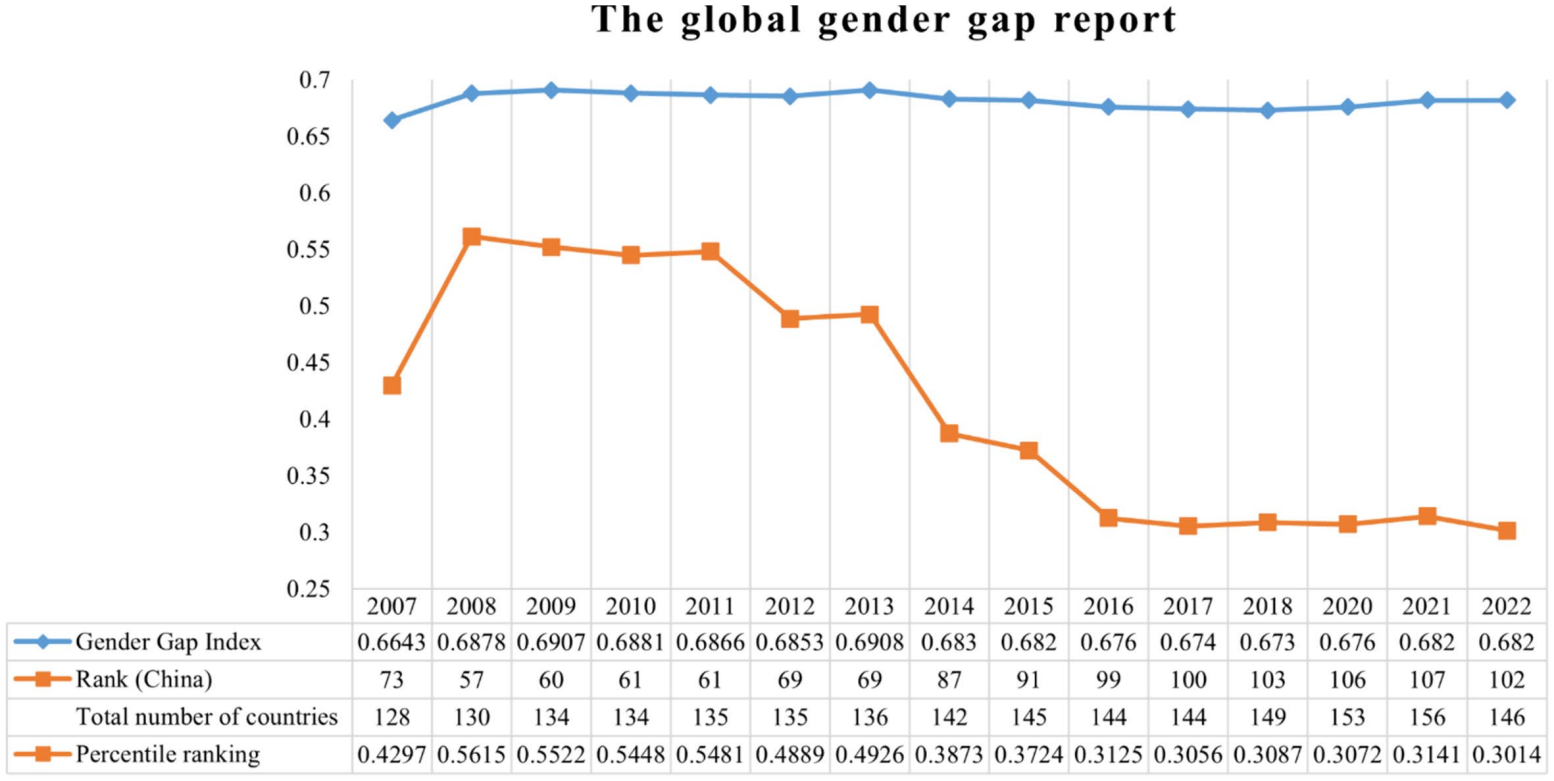
The global gender gap report.

Gender equality is a basic state policy of China and one of the daily issues of the United Nations. As an important goal for social progress and human sustainable development, gender equality has been highly valued by China (Yang, 2021). At the 18th National Congress of the Communist Party of China, China included gender equality as a basic state policy in its report for the first time (Sun, 2023). It was clearly stated that men and women enjoy equal rights and shoulder equal obligations in politics, economics, culture, society, the family and other aspects (Sun, 2023). Nevertheless, the gender gap in China is striking. According to the “Global Gender Gap Report 2022” released by the World Economic Forum (World Economic Forum, 2022), China ranks 102nd out of 146 countries (Figure 1). Since 2011, perhaps due to the gradual liberalization of the two-child policy, China’s gender equality index has increased but its percentile ranking still shows a sharp decline against the background of globalization. Identifying ways to promote gender equality remains an urgent social problems in China.

A high level of gender equality can improve the physical and mental health and social development of both men and women, leading to generally higher levels of happiness, life satisfaction, and mental health and less depression (Holter, 2014; Looze et al., 2018; Heinz et al., 2020). The higher the level of gender equality in a country or region, the longer the life expectancy of both men and women (Kolip and Lange, 2018; Kolip et al., 2019) and the lower the violent death rate and suicide rate of both men and women (Milner et al., 2020). At the same time, gender inequality has certain negative effects on social and economic development (Lippa et al., 2010). Studies show that the gender inequality index is inversely proportional to the gross national product and proportional to the resident income gap (Wells et al., 2012). In addition, gender equality has positive communication and social integration effects that can reduce the atmosphere of social violence caused by the societal preference for sons (Saeed Ali et al., 2017).

Consciousness of gender equality, which belongs to the interdisciplinary research field of sociology and psychology, is the opposite of gender stereotypes. It refers to people’s perceptions of and attitudes toward equal rights and obligations of men and women in various fields, including respect for equal rights of men and women in various areas of society, family and personal life (Su et al., 2020). Studies have found that consciousness of gender equality can help boys and girls eliminate stereotypes and reduce depression while promoting their mental health (Zhao, 2018). At the same time, it can also promote the development of social equality and improve social fairness by influencing behavioral consciousness, which is of great importance for the construction of a society with advanced views of gender (Lin and Ye, 2015).

Most adults’ consciousness of gender equality is strongly influenced by their childhood, especially during school age. This refers to the period from primary school to puberty, usually from 6 or 7 years old to 12 or 13 years old (Wang, 2013), which is a critical time for the development of and changes to individual gender consciousness (Banse et al., 2010; Siyanova-Chanturia et al., 2015).

Trautner et al. (2005) suggested that individual gender stereotypes start to appear at the age of 5–6, reach a peak of stereotypical rigidity at the age of 7–8, and then become more flexible. Zhao et al. (2020) found that gender stereotypes emerge in children aged 6–10, with significant differences in different genders. Stereotypes are usually more positive for the same gender group and more negative for different gender groups. Su et al. (2020) found that children’s consciousness of gender equality gradually increases with age. Girls’ consciousness of gender equality is generally higher than that of boys, and both sexes have the lowest consciousness of gender equality in relation to occupation. Spanish scholars’ research on the occupational gender stereotypes of children found that occupational gender stereotypes appear in children aged 4–9, and children of different genders and ages have different gender stereotypes of male and female occupations. Solbes-Canales et al. (2020) found that found that ages 6–7 are the turning point for boys’ and girls’ cognition of masculine occupational stereotypes.

School-aged children already have gender knowledge and awareness. Gender stereotypes are not conducive to their healthy growth. However, due to the immature mental development of children at this stage, these stereotypes are in a dynamic state of development and may change due to the influence of family, education or peers. Therefore, an in-depth exploration of the factors that affect the consciousness of gender equality of school-aged children may be helpful to cultivate children’s consciousness of gender equality.

### 1.1 Theories and influencing factors in the formation of children’s consciousness of gender equality

Gender consciousness is a crucial aspect of human self-awareness. Various scholars have proposed different theoretical hypotheses for the formation of children’s gender consciousness based on different research perspectives, including biosocial theory (Wood and Eagly, 2002; Shang, 2019), cognitive development theory (Xing, 2002), gender schema theory (Cann and Newbern, 1984; Pillow et al., 2022), and social learning theory (Bussey and Bandura, 1984). These theories comprehensively explore the factors that influence the formation and development of children’s gender consciousness from multiple perspectives and can generally be categorized into three main aspects: individual factors, psychological cognitive factors, and social environmental factors.

#### 1.1.1 Individual factors

According to biosocial theory (Wood and Eagly, 2002; Shang, 2019), differences in gender consciousness between men and women result from the combined effects of physiological differences and the division of social roles. Cognitive development theory (Xing, 2002) posits that children’s gender consciousness develops continuously with age as their cognitive abilities mature. Based on these two theories, we believe that the main individual factors that affect school-aged children’s consciousness of gender equality are gender and age, as confirmed by previous studies (Siyanova-Chanturia et al., 2015). A study by Tang et al. (2011) confirmed that girls are more aware than boys of gender equality at all school levels. Solbes-Canales et al. (2020) analyzed the consciousness of occupational gender equality among children aged 4–9 years and found that although occupational gender stereotypes are common among school-aged children, the consciousness of occupational gender equality increases with age for both boys and girls. Research by Siyanova-Chanturia et al. (2015) on consciousness of gender equality across different age groups also showed that age and gender are crucial factors that influence the development of consciousness of gender equality in school-aged children.

#### 1.1.2 Psychological cognitive factors

According to gender schema theory (Cann and Newbern, 1984; Pillow et al., 2022), children’s gender consciousness develops primarily through the continuous enrichment and accumulation of various gender information schemas, such as associating pink with girls and blue with boys. The greater the differentiation in children’s gender schemas, the stronger their gender role stereotypes (Chen, 2016). Gender roles serve as indicators that reflect the degree of differentiation in children’s gender schemas and are important psychological cognitive factors that influence children’s consciousness of gender equality.

The American psychologist Bem (1974) proposed the theory of gender role androgyny, which categorizes individual gender roles into four types: masculine, feminine, undifferentiated, and androgynous. A masculine gender role refers to the set of societal expectations, behaviors, and characteristics traditionally associated with being male (Bem, 1974). These roles typically emphasize attributes such as assertiveness, strength, independence, competitiveness, and emotional restraint. A feminine gender role refers to the set of societal expectations, behaviors, and characteristics traditionally associated with being female (Bem, 1974). These roles typically emphasize attributes such as nurturing, empathy, sensitivity, cooperation, and emotional expressiveness. An undifferentiated gender role refers to a lack of strong identification with traditional masculine or feminine characteristics and behaviors (Bem, 1974). Individuals with undifferentiated gender roles do not exhibit distinct traits or behaviors typically associated with either gender. An androgynous gender role refers to a combination of both masculine and feminine characteristics and behaviors within a single individual (Bem, 1974). People who identify with androgynous gender roles can exhibit traits traditionally associated with both genders, such as assertiveness and nurturing, independence and empathy.

Bem (1974) suggested that in an ideal world, individuals, regardless of gender, would embody both male and female character traits and exhibit androgynous gender roles. Numerous studies (Geng and Zhang, 2012; Yu et al., 2020) have confirmed that individuals with androgynous gender roles tend to have better self-esteem, subjective well-being, and social adaptability because they possess the positive qualities of both sexes. Deng (2016) also found that children with higher consciousness of gender equality are more likely to exhibit androgynous gender roles.

#### 1.1.3 Social and environmental factors

Bandura’s social learning theory (Bussey and Bandura, 1984; Tang et al., 2011) posits that children acquire their consciousness of gender equality through imitative learning from parents, teachers, and peers in their environment. Studies show that the higher the consciousness of gender equality of parents is, the higher the consciousness of gender equality of their children (Tenenbaum and Leaper, 2002). Additionally, school-based education that promotes gender role androgyny can significantly reduce gender stereotypes among school-aged children, thereby fostering their consciousness of gender equality (Guo et al., 2014). Interaction with peer groups through play also influences the formation of children’s gender stereotypes and their consciousness of equality (Li and Lin, 2021). Moreover, an environment that strongly promotes gender equality is conducive to the development of children’s consciousness of gender equality (Zhao, 2021).

In conclusion, numerous studies underscore the pivotal roles of individual, psychological, and social environmental factors in shaping children’s gender equality consciousness. However, existing studies often lack a cohesive theoretical framework to comprehensively analyze the intricate interplay among these elements. Therefore, establishing a robust macro-theoretical framework is a promising way to illuminate the extent and pathways of interaction among these factors. This endeavor is important for refining effective educational strategies to promote gender equality among children.

### 1.2 Ecosystem theory

The theoretical model of the ecosystem, proposed by Bronfenbrenner in 1979, emphasizes that individuals develop within environmental systems (Bronfenbrenner and Ceci, 1994) that interact with individuals and influence their development. The model includes four layers, the microsystem, mesosystem, exosystem, and macrosystem, which represent the direct to indirect influences of different environments on individuals. The microsystem consists of environments that are in direct contact with individuals and have the most immediate impact, such as family, school, peer groups, and networks. The mesosystem encompasses the relationships and processes between individuals and their microsystems, such as parent–child relationships, teacher-student interactions, and peer relationships. The exosystem includes environments that individuals are not directly involved in but that affect their development, such as parents’ workplaces. Finally, the macrosystem refers to the broader cultural and social context. These four systems complement each other and collectively influence children’s development.

**Figure 2:**
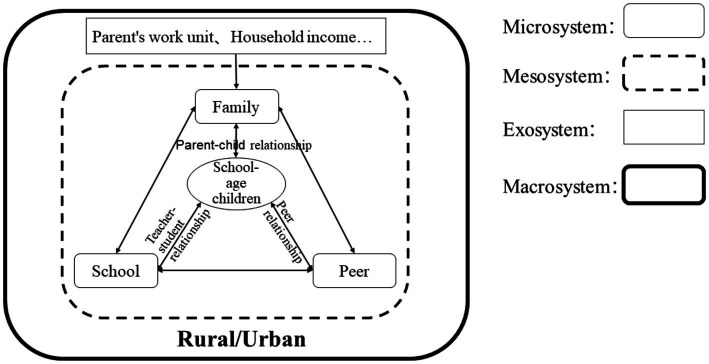
Theoretical model.

**Figure 3:**
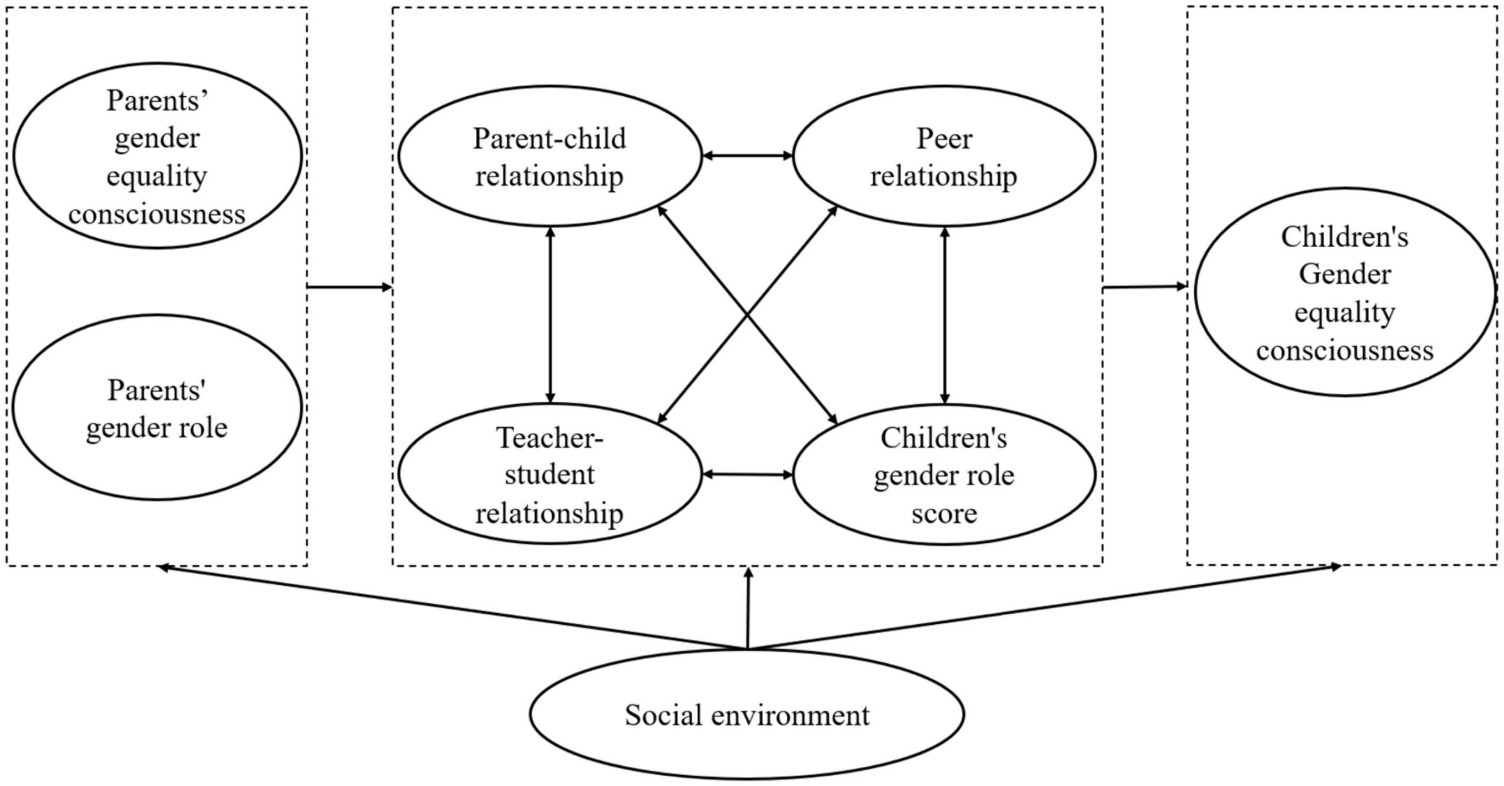
Theoretical framework.

According to the study by Paul-Halpern and Perry-Jenkins (2016), family has a profound and important influence on children’s consciousness of gender equality. Therefore, from the perspective of children’s family environment and based on ecosystem theory, this study focuses on school-aged children and introduces three factors that may have the most direct impact on their consciousness of gender equality: school, family and peers. We take the teacher-student relationship, the parent–child relationship and peer relationships as the mesosystem, the parents’ work unit and household income as the exosystem, and the different social and cultural environments of rural and urban areas in China as the macrosystem to construct the ecosystem model (Figure 2) and the schematic diagram of the influence process (Figure 3) of this study.

### 1.3 Aims

This study aims to understand school-aged children’s consciousness of gender equality. We consider the influencing factors and their action paths in a cross-sectional investigation that uses multi-group structural equation modeling to comprehensively explore the path model of consciousness of gender equality among school-aged children. In doing so, we provide a corresponding theoretical basis and countermeasures for the family and school to conduct follow-up education on gender equality for school-aged children.

## 2 Methods

### 2.1 Design and sample

In this cross-sectional study, two urban primary schools and two rural schools were randomly selected by the computer system of the Hunan Education Bureau from June 2021 to September 2021. Primary school students in grades 1–6 who met the inclusion and exclusion criteria and their parents were selected as the research subjects to complete a questionnaire survey. The study followed the principle of voluntary participation, and informed consent was obtained from all participating children and their guardians before the investigation began.

The inclusion criteria were as follows: (1) school-aged children (Wang, 2013) (aged 6–13) in grades 1–6 and their parents (2) who provided informed consent and voluntary participation. The exclusion criteria were (1) primary school students with cognitive impairment or major mental illness and (2) people who had previously participated in the same type of research.

This study was conducted in June 2020 in Changsha City at Guijing Elementary School. School-age children’s consciousness of gender equality was examined. Use the sample standard deviation (*σ* = 8.387) to represent the population standard deviation, allowable error was not more than 0.5, *α* = 0.05, using the overall mean with the required sample size estimation formula *n* = (u_α/2_σ/δ)^2^. The results suggested a sample size of 1,085. Considering 20% invalid questionnaires, we needed to survey at least 1,302 pupils.

A total of 1,500 questionnaires were distributed and 1,312 usable responses were obtained, corresponding to a return rate of 87.5%.

### 2.2 Data collection

A questionnaire was conducted from June 2021 to September 2021. The study adhered to the principle of voluntary participation, and informed consent was obtained from all participating schools, children, and guardians before the investigation began. All questionnaire items were written in Chinese Pinyin. For students below grade 3, data collectors were trained in advance. While the participants completed the questionnaire, the data collectors assisted lower-grade students by reading the words aloud to ensure accurate responses. To ensure the reliability of the survey instrument, a pilot test was conducted with 200 children in June 2020 at Guijing Elementary School in Changsha. The scales were confirmed to be applicable based on the results of the pilot test.

### 2.3 Measurements

The consciousness of gender equality questionnaire and gender role scale were to be completed by both children and parents. The demographic and gender-related characteristics questionnaire, parent–child compatibility questionnaire, teacher-student relationship questionnaire, and peer relationship subscale were to be completed by children only. The family-related characteristics questionnaire was to be completed by parents only.

#### 2.3.1 Questionnaire for both children and parents

##### 2.3.1.1 Consciousness of gender equality questionnaire

The consciousness of gender equality questionnaire was developed by Tang et al. (2011). The questions addressed attitudes toward gender equality in three domains: family (e.g., “Who do you think should do the cooking?”), school (e.g., “Who do you think should be given more chances by the teacher to act as a monitor?”), and occupation (e.g., “Who do you think is more suitable to be a kindergarten teacher?”). Each domain contained 10 items for a total of 30 items. For scoring, option B (“same for men and women”) was coded as 1, while options A (“more suitable for men”) and C (“more suitable for women”) were both coded as 0. A higher total score indicated a stronger consciousness of gender equality. This scale has been found to be suitable for people of all ages, including children and adults (Tang et al., 2011). The questionnaire demonstrated high reliability with a Cronbach’s *α* of 0.92.

##### 2.3.1.2 Gender role scale

The Bem Sex Role Inventory (BSRI) was designed by Bem (1974) and translated by Lu and Su (2003). It measures gender role orientation using a 7-point Likert-type scale where 1 represents “never true” and 7 represents “always true.” The BSRI consists of three subscales: a 14-item masculinity scale (e.g., ambitious and aggressive), a 12-item femininity scale (e.g., gentle and affectionate), and a 20-item gender-neutral scale (e.g., helpful and happy). The total score of the masculinity scale is divided by 14, and the total score of the femininity scale is divided by 12. If both scores exceed four, the result is considered androgynous. If only the masculinity score exceeds four, the result is masculine; if only the femininity score exceeds four, the result is feminine. If both scores are less than four, the result is undifferentiated. This scale has been proven to be suitable for people of all ages, including children and adults (Wu and Wu, 2012). The BSRI has an internal consistency and test–retest reliability of approximately 0.80. Cronbach’s *α* was 0.91 for this study.

#### 2.3.2 Questionnaire for children only

##### 2.3.2.1 Demographic and gender-related characteristics

This section included gender, age, grade, place of residence, whether the participant was an only child, whether the father or mother worked outside the home, parents’ feelings, parental quarrels, the number of same-sex friends, the number of opposite-sex friends, and friend-making tendencies.

##### 2.3.2.2 Parent–child compatibility questionnaire

The parent–child compatibility questionnaire compiled by Olson et al. (1979) and revised by Wang and Zhang (2007) was adopted for measurement in this study. The questionnaire includes two parts, paternal affinity and maternal affinity, which are used to measure the degree of parental affinity. The questionnaire contains 20 items in total, and each sub-questionnaire consists of 10 items. It uses a 5-point Likert-type scale where 1 represents “never” and 5 represents “always.” The higher the score on the questionnaire, the better the parent–child relationship. The scale has been found to have good reliability and validity in Chinese primary school students (Li et al., 2023). The total reliability of the questionnaire was 0.82, the internal consistency coefficient of the parent–child affinity questionnaire was 0.79, and the internal consistency coefficient of the parent–child affinity questionnaire was 0.82. Cronbach’s α was 0.84 for this study.

##### 2.3.2.3 Teacher-student relationship questionnaire

This study adopted the teacher-student relationship questionnaire revised by Zhang (2003) for measurement. The questionnaire consists of 22 items, including four dimensions of conflict, avoidance, intimacy and attachment. Each student was required to assess the degree of compliance with the described situation based on his or her daily relationship with the teacher. The scale uses a 5-point Likert-type scale where 1 represents “never” and 5 represents “always.” The higher the total score is, the better the teacher-student relationship. The scale has been found to have good reliability and validity in Chinese primary school students. Cronbach’s *α* was 0.83 for the Chinese version and 0.64 for this study.

##### 2.3.2.4 Peer relationship subscale

This study adopted the peer relationship subscale of the self-description questionnaire modified by Marsh et al. (1984) and Chen et al. (1997). This scale includes 10 items that are scored on a 6-point Likert-type scale ranging from 1 (“strongly disagree”) to 6 (“strongly agree”). The higher the total score, the better the peer relationship. The scale has been found to have good reliability and validity in Chinese primary school students (Chen et al., 1997). The subscale is widely used to measure children’s peer relationships. Cronbach’s *α* was 0.70 for this study.

#### 2.3.3 Questionnaire for parents only

##### 2.3.3.1 Family-related characteristics

This section included information on family monthly income, family income source, father’s education level, and mother’s education level.

### 2.4 Ethical considerations

Before data collection, ethics approval was obtained from the ethics committee of our university (No. E202165). Permission to collect data was granted by the principal and head teacher at each school before the survey was conducted. The participants were informed of the purpose, method, and considerations of the study and were told that they could quit at any time while completing the survey. The students and their parents or legal guardian(s) signed an informed consent form. The cover page of the questionnaire contained contact information for psychological consultations if the participants needed them.

### 2.5 Data analysis

SPSS26.0 and AMOS24.0 software were used in this study for statistical analysis. The frequency, percentage, and mean ± standard difference were used to describe the general demographic data of the school-aged children, the children’s consciousness of gender equality and the status of influencing factors. An independent-sample *t-*test and one-way analysis of variance were used to determine the differences in the demographic data, children’s gender roles, parents’ gender roles and consciousness of gender equality of school-aged children. The LSD test was used to compare the differences among multiple groups. Pearson correlation analysis was used to clarify the correlation between the consciousness of gender equality of school-age children and children’s gender role score, parent–child relationships, teacher-student relationships, peer relationships, parents’ gender role score and parents’ consciousness of gender equality. Multiple stepwise linear regression was used to analyze the relevant factors that affected the consciousness of gender equality of school-age children. The steps described above helped us define the main factors that influenced school-age children’s consciousness of gender equality. The structural equation model was used to analyze the interaction path and intensity of each influencing factor. Multi-group structural equation model was used to analyze the similarities and differences of the consciousness of gender equality pathway model among school-aged children in different groups. The above steps helped to clarify how the family environment influences the consciousness of gender equality of various groups of children.

## 3 Results

### 3.1 Participant demographics

In this study, there were 645 school-aged boys and 667 girls, with a male to female ratio of 0.97:1. The mean age of the children was (10.02 ± 1.83) years. The mean age of the parents was (37.96 ± 7.43) years. There were 419 male parents, accounting for 31.9%, and 893 female parents, accounting for 68.1%. Among the parents, 386 were the father of the child, accounting for 29.4%, while 866 were the mother of the child, accounting for 66.0%; 60 were other parents, accounting for 4.6%. The frequency and percentage of use were used to describe the general demographic data of the school-aged children (Table 1).

**Table 1:** Relationships among socio-demographic characteristics and variable scores (*n* = 1,312).

Variables		*N*	Gender equality consciousness	*t*/*F*	*P*	Pairwise comparison	*P*
Gender	Male	645	16.69 ± 7.75	−3.518	0.000^*^		
	Female	667	18.19 ± 7.67				
Age	6	8	18.13 ± 8.24	16.667	0.000^*^	a < c	0.000^*^
						a < d	0.009^*^
	7^a^	162	13.86 ± 7.83			a < e	0.000^*^
						a < f	0.000^*^
	8^b^	161	15.36 ± 7.45			a < g	0.000^*^
						b < c	0.046^*^
	9^c^	166	17.01 ± 7.22			b < e	0.000^*^
						b < f	0.000^*^
	10^d^	201	15.93 ± 7.30			b < g	0.000^*^
						c < e	0.033^*^
	11^e^	247	18.60 ± 7.66			c < f	0.000^*^
						c < g	0.016^*^
	12^f^	315	20.28 ± 7.25			d < e	0.000^*^
						d < f	0.000^*^
	13^g^	52	19.85 ± 7.24			d < g	0.001^*^
						e < f	0.008^*^
Grade	Grade1^a^	189	13.87 ± 8.00	29.277	0.000^*^	a < b	0.001^*^
	Grade2^b^	166	16.48 ± 6.83			a < c	0.003^*^
						a < d	0.014^*^
	Grade3^c^	186	16.10 ± 7.70			a < e	0.000^*^
						a < f	0.000^*^
	Grade4^d^	231	15.65 ± 7.21			b < e	0.000^*^
						b < f	0.000^*^
	Grade5^e^	238	20.20 ± 7.28			c < e	0.000^*^
						c < f	0.000^*^
	Grade6^f^	302	20.29 ± 7.15			d < e	0.000^*^
						d < f	0.000^*^
Residence	Urban	609	17.64 ± 7.73	0.804	0.422		
	Rural	703	17.30 ± 7.75				
The only child	Yes	199	16.87 ± 7.84	−1.161	0.246		
	No	1,113	17.56 ± 7.72				
The left-behind children	Yes	326	16.01 ± 7.56	−3.914	0.000^*^		
	No	986	17.93 ± 7.74				
Parents’ emotional status	Good^a^	841	18.12 ± 7.59	7.875	0.000^*^	b < a	0.005^*^
	Average^b^	339	16.73 ± 7.88			c < a	0.000^*^
	Bad^c^	57	14.07 ± 8.55			d < a	0.015^*^
	Divorce^d^	75	15.87 ± 7.01			c < b	0.016^*^
Parental quarrel	Never^a^	554	18.03 ± 7.91	6.948	0.001^*^	c < a	0.000^*^
	Few^b^	672	17.33 ± 7.41			c < b	0.003^*^
	Many^c^	86	14.74 ± 8.57				
Number of same -sex friends	0	34	15.50 ± 7.70	3.171	0.024^*^		
	1^a^	96	15.89 ± 8.53				
	2–3	201	16.83 ± 7.69			a < b	0.020^*^
	>3^b^	981	17.81 ± 7.65				
Number of opposite-sex friends	0	321	17.91 ± 7.45	2.480	0.060		
	1	219	16.19 ± 7.54				
	2–3	312	17.72 ± 7.47				
	>3	460	17.56 ± 8.17				
Friend-making tendency	Same -sex friends	1,153	17.69 ± 7.60	2.709	0.007^*^		
	Opposite-sex friends	159	15.76 ± 8.52				
Family monthly income	0–1,000^a^	89	18.08 ± 8.83	3.741	0.005^*^	b < a	0.036^*^
	1,001–2000^b^	247	16.08 ± 7.04			b < c	0.024^*^
	2001–3,000^c^	336	17.54 ± 7.36			b < d	0.000^*^
	3,001–5,000^d^	382	18.40 ± 7.83			e < d	0.029^*^
	>5,001^e^	258	17.04 ± 8.16				
Family income source	Farming	27	15.78 ± 7.02	1.035	0.376		
	Business	234	17.32 ± 7.69				
	Employee	826	17.37 ± 7.86				
	Other	225	18.12 ± 7.40				
Father’s education level	Primary school and below^a^	77	16.04 ± 8.51	2.929	0.033^*^	a < b	0.050^*^
	Junior high school^b^	455	17.91 ± 7.61			d < b	0.030^*^
	Senior high school^c^	391	17.91 ± 7.76			d < c	0.036^*^
	University or above^d^	389	16.75 ± 7.85				
Mother’s education level	Primary school and below	95	16.96 ± 8.03	4.177	0.006^**^	c < a	0.008^*^
	Junior high school^a^	443	17.82 ± 7.81			c < b	0.001^*^
	Senior high school^b^	389	18.22 ± 7.26				
	University or above^c^	385	16.39 ± 7.96				
Children’s gender role	Masculinity^a^	81	15.91 ± 6.93	6.131	0.000^*^	a < d	0.017^*^
	Femininity^b^	184	15.86 ± 7.94			b < d	0.000^*^
	Undifferentiated^c^	137	16.56 ± 8.26			c < d	0.035^*^
	Androgynous^d^	910	18.05 ± 7.62				
Parents’ gender role	Masculinity^a^	30	14.57 ± 8.02	3.937	0.008^*^		
	Femininity^b^	135	16.27 ± 7.87			a < c	0.024^*^
	Undifferentiated	88	16.19 ± 8.23			b < c	0.030^*^
	Androgynous^c^	1,059	17.79 ± 7.64				

^*^P < 0.05. Superscript letters “a” to “d” are used to distinguish different variable groups.

### 3.2 Consciousness of gender equality among school-aged children

The total score for the consciousness of gender equality of school-aged children was (17.46 ± 7.74) points, including (6.62 ± 2.77) points in family field, (6.13 ± 3.00) points in the school field and (4.71 ± 3.10) points in the occupational field. The total scores were 18.19 ± 7.67for girls’ consciousness of gender equality, 6.81 ± 2.76 for family, 6.41 ± 2.94 for school, and 4.97 ± 3.06 for career. The total score for male students was 16.69 ± 7.75 for gender equality, 6.42 ± 2.77 for family, 5.83 ± 3.03 for school, and 4.44 ± 3.11 for career. The total score for the consciousness of gender equality of school-aged boys and girls and their scores in various dimensions are shown in Table 2.

**Table 2:** Descriptive statistics of the measured variables (*n* = 1,312).

Variables	Items	Scores		
		Boy	Girl	All
Family fields	10	6.42 ± 2.77	6.81 ± 2.76	6.62 ± 2.77
School fields	10	5.83 ± 3.03	6.41 ± 2.94	6.13 ± 3.00
Occupational fields	10	4.44 ± 3.11	4.97 ± 3.06	4.71 ± 3.10
Gender equality consciousness	20	16.69 ± 7.75	18.19 ± 7.67	17.46 ± 7.74

**Figure 4:**
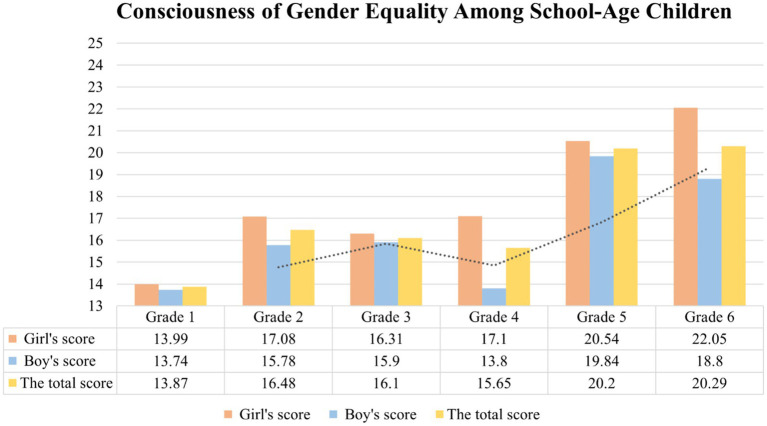
Gender equality consciousness of school-age children.

The change trend of consciousness of gender equality of school-aged boys and girls of different grades is shown in Figure 4.

### 3.3 Univariate analysis of influencing factors on the consciousness of gender equality among school-aged children

The univariate analysis showed that the consciousness of gender equality of school-aged children was statistically significant in terms of gender, age, grade, parents’ migrant work, parents’ relationship status, parents’ quarrels, who they lived with, the number of same-sex friends, friend-making intention, family monthly income, father’s education level, mother’s education level, the child’s gender role and the parent’s gender role (*p* < 0.05) (Table 1).

### 3.4 Correlation analysis of factors influencing the consciousness of gender equality among school-aged children

Table 3 shows that the correlation analysis of the consciousness of gender equality of school-aged children and its influencing factors found that the consciousness of gender equality of school-aged children was significantly positively correlated with their gender role scores, parent–child relationship, teacher-student relationship, parents’ consciousness of gender equality and parents’ gender role scores (*p* < 0.05).

**Table 3:** Correlations among the measured variables (*n* = 1,312).

Variables	1	2	3	4	5	6	7
1. Children’s gender equality consciousness	1						
2. Children’s sex role score	0.143^*^	1					
3. Parent–child relationship	0.161^*^	0.465^*^	1				
4. Teacher-student relationship	0.179^*^	0.358^*^	0.424^*^	1			
5. Peer relationship	0.050	0.390^*^	0.343^*^	0.358^*^	1		
6. Parents’ gender equality consciousness	0.557^*^	0.119^*^	0.155^*^	0.155^*^	0.055^*^	1	
7. Parents’ gender role score	0.148^*^	0.511^*^	0.317^*^	0.312^*^	0.262^*^	0.175^*^	1

^*^P < 0.05.

### 3.5 Multiple stepwise linear regression analysis of consciousness of gender equality among school-aged children

Multiple stepwise linear regression analysis was conducted. The consciousness of gender equality of school-aged children was used as the dependent variable, and significant variables from the univariate analysis and the correlation analysis were used as the independent variables. The unordered categorical variables (a variable that has no order and no hierarchy but can be classified and counted), parents’ feeling, children’s gender role, and parents’ gender role, were converted into dummy variables.

Children’s age, gender, gender role, parent–child relationship, teacher-student relationship and parents’ consciousness of gender equality were entered into the regression equation. The absolute value of the standardization coefficient of parents’ consciousness of gender equality was the largest, indicating that parents’ consciousness of gender equality had the greatest impact on school-aged children’s consciousness of gender equality. The complex correlation coefficient *R* = 0.629, the determination coefficient R2 = 0.395, the adjusted R2 = 0.392, and the overall test *F* value of the regression model = 142.092 (*p* = 0.000). Therefore, the overall explanatory variation of the regression model reached a significant level, and these 7 variables could explain 39.5% of the variation of the dependent variable. The regression equation of factors that influenced the consciousness of gender equality of school-aged children is as follows: *Y* = −14.350 + 0.524×1 + 1.083×2 + 0.065×3 + 0.997×4 + 1.030×5 + 0.037×6 (X1 = parents’ consciousness of gender equality, X2 = age, X3 = teacher-student relationship, X4 = children’s androgynous gender role, X5 = gender, X6 = parent–child relationship) (Table 4).

**Table 4:** Multiple linear regression analysis of the study variables on the consciousness of gender equality (*n* = 1,312).

Variables	*B*	SE	*β*	*t*	*P*
(Constant)	−14.350	1.815		−7.904	0.000^*^
Parents’ gender equality consciousness	0.524	0.023	0.506	22.947	0.000^*^
Age	1.083	0.089	0.270	12.133	0.000^*^
Teacher-student relationship	0.065	0.016	0.104	4.169	0.000^*^
Children’s gender role: androgynous	0.997	0.391	0.059	2.550	0.11^*^
Gender	1.030	0.339	0.067	3.036	0.002^*^
Parent–child relationship	0.037	0.016	0.058	2.353	0.019^*^

*R*^2^ = 0.395, *F* = 142.092, *P* = 0.000; ^*^P < 0.05.

### 3.6 Structural equation model analysis of factors influencing consciousness of gender equality among school-aged children

**Figure 5:**
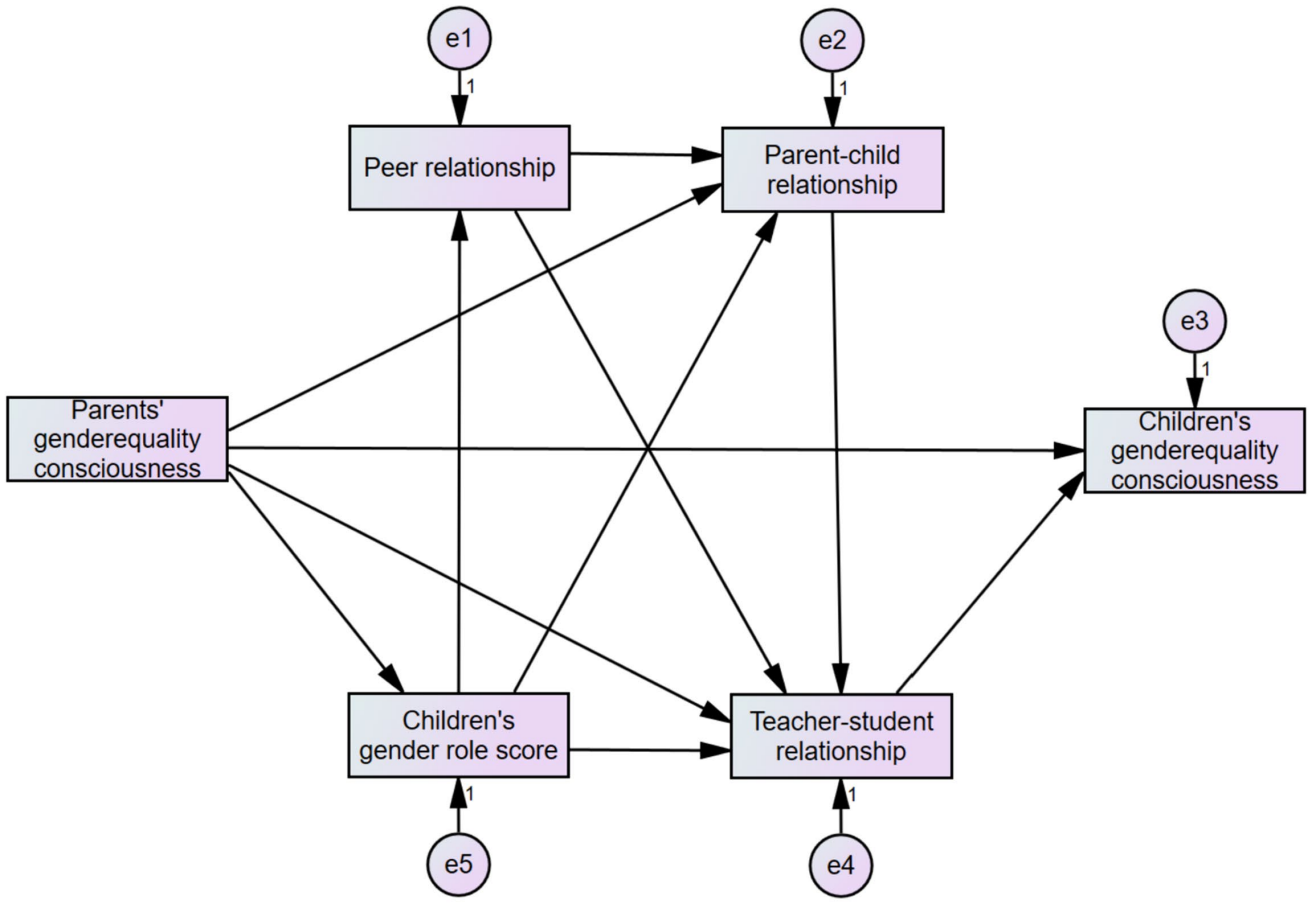
Initial model M1.

Based on the relevant literature and theoretical basis and combined with the above analysis results, this study constructed the initial model M1 of the factors that influenced the consciousness of gender equality of school-aged children (Figure 5).

**Figure 6:**
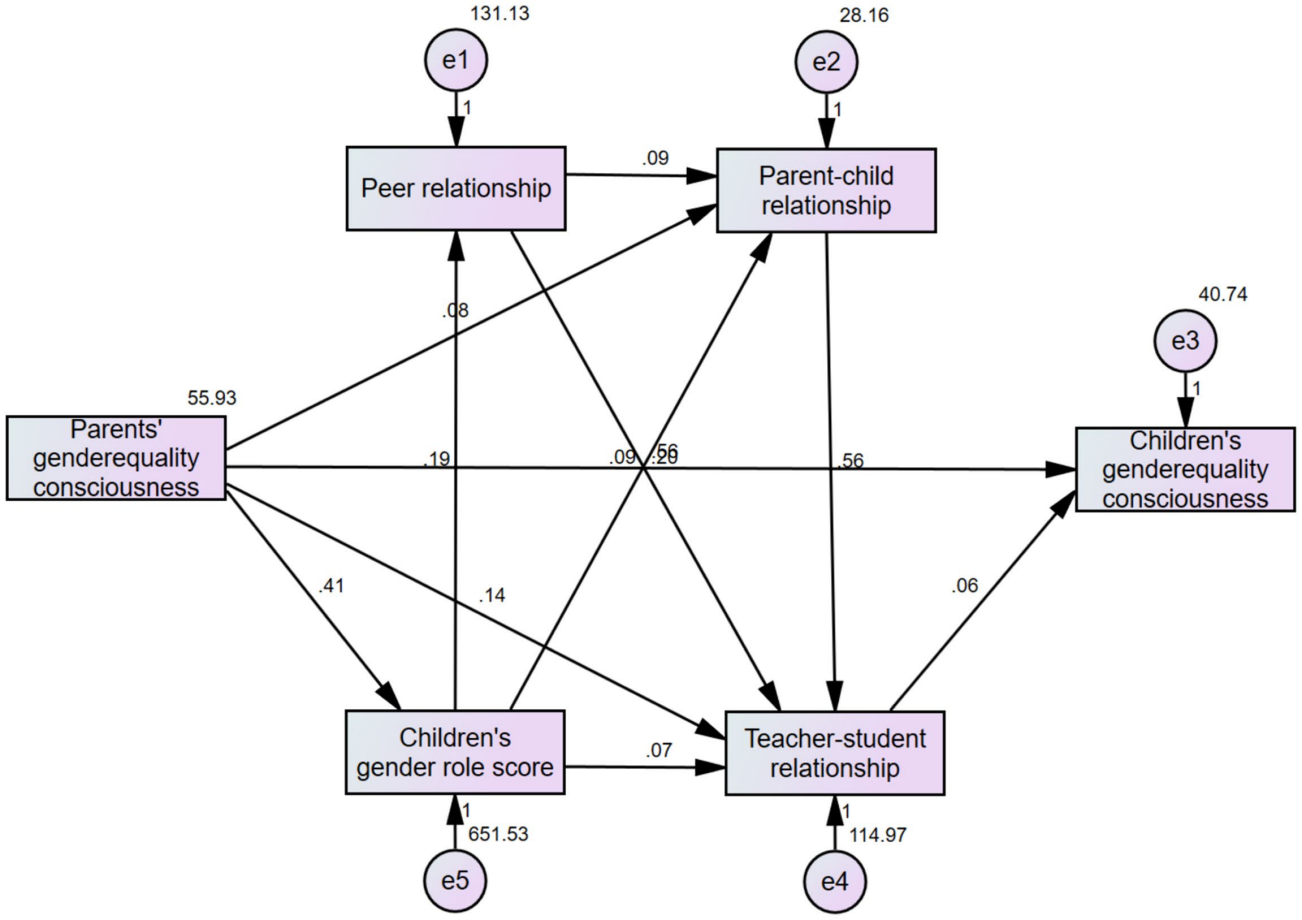
Modified model M2.

After testing the initial structural equation model and deleting the non-statistically significant path, a modified model M2 with good model fit was obtained (Figure 4). The parameters of the model were as follows: chi-square = 8.011, DF = 4, 1 < chi square/DF = 2.003 < 3, *p* = 0.091 > 0.05, GFI = 0.998 > 0.9, NFI = 0.995 > 0.9, IFI = 0.997 > 0.9, TLI = 0.990 > 0.9, CFI = 0.997 > 0.9, RMSEA = 0.028 < 0.05. The model fit well (Wu, 2010). The standardized parameters of the final model are shown in Figure 6. Table 5 shows the model’s path relationships. Table 6 shows the direct effect, indirect effect and total effect among the factors.

**Table 5:** Maximum likelihood estimates of the modified model (*n* = 1,312).

Pathway	Non-standardized coefficients	Standardized coefficients	SE	C.R.	*P*
Children’s gender equality consciousness ← Parents’ gender equality consciousness	0.561	0.543	0.024	23.527	0.000^*^
Children’s gender equality consciousness ← Teacher-student relationship	0.059	0.094	0.014	4.095	0.000^*^
Children’s sex role score ← Parents’ gender equality consciousness	0.411	0.119	0.094	4.357	0.000^*^
Peer relationship ← Children’s sex role score	0.189	0.390	0.012	15.351	0.000^*^
Parent–child relationship ← Children’s sex role score	0.091	0.379	0.006	14.562	0.000^*^
Parent–child relationship ← Peer relationship	0.094	0.189	0.013	7.324	0.000^*^
Parent–child relationship ← Parents’ gender equality consciousness	0.081	0.099	0.020	4.118	0.000^*^
Teacher-student relationship ← Parents’ gender equality consciousness	0.140	0.084	0.040	3.486	0.000^*^
Teacher-student relationship ← Children’s sex role score	0.067	0.140	0.014	4.973	0.000^*^
Teacher-student relationship ← Peer relationship	0.204	0.204	0.026	7.713	0.000^*^
Teacher-student relationship ←Parent–child relationship	0.556	0.276	0.056	9.954	0.000^*^

^*^P < 0.05.

**Table 6:** Standardized direct, indirect, and total effects for the modified model (*n* = 1,312).

Dependent variable	Effect	Independent variable				
		Parents’ gender equality consciousness	Children’s sex role score	Peer relationship	Parent–child relationship	Teacher-student relationship
Children’s sex role score	Direct effect	0.119				
	Indirect effect	0.000				
	Total effect	0.119				
Peer relationship	Direct effect	0.000	0.390			
	Indirect effect	0.047	0.000			
	Total effect	0.047	0.390			
Parent–child relationship	Direct effect	0.099	0.379	0.189		
	Indirect effect	0.054	0.074	0.000		
	Total effect	0.153	0.453	0.189		
Teacher-student relationship	Direct effect	0.084	0.140	0.204	0.276	
	Indirect effect	0.068	0.205	0.052	0.000	
	Total effect	0.153	0.344	0.256	0.276	
Children’s gender equality consciousness	Direct effect	0.543	0.000	0.000	0.000	0.094
	Indirect effect	0.014	0.033	0.024	0.026	0.000
	Total effect	0.557	0.033	0.024	0.026	0.094

### 3.7 Multi-group structural equation model analysis of factors influencing consciousness of gender equality among school-aged children

According to the previous analysis, gender and age were the main demographic variables that affected consciousness of gender equality among school-aged children. In this study, most children aged 6–8 years old were in the first and second grade, children aged 9–10 years old were in the third and fourth grade, and children aged 11–13 years old were in the fifth and sixth grade. The preliminary analysis results showed that the consciousness of gender equality of the first and second grade students and the fifth and sixth grade students showed an upward trend and stable development, while the consciousness of gender equality of the third and fourth grade students showed a slight fluctuation. Considering the feasibility of the follow-up implementation of gender equality education, we decided to classify grades 1 and 2 as the lower study period, grades 3 and grade 4 as the middle study period, grades 5 and 6 as the higher study period. We used a multi-group structural equation model for further analysis.

Table 7 shows the path difference of the structural equation model of the consciousness of gender equality of children of different genders. There was no significant difference between the different constrained models [ΔP(measurement weights) = 0.26 > 0.05, ΔP(measurement covariances) = 0.10 > 0.05, ΔNFI(measurement residuals) = −0.026 < 0.05, ΔIFI(measurement residuals) = −0.015 < 0.05, TLI(measurement residuals) = −0.011 < 0.05, RFI(measurement residuals) = −0.01 < 0.05]. Therefore, the model was applicable to school-aged children of different genders.

**Table 7:** Multi-group analysis across gender (*n* = 1,312).

Pathway	Boys					Girls				
	Non-standardized coefficients	Standardized Coefficients	SE	C.R.	*P*	Non-standardized coefficients	Standardized coefficients	SE	C.R.	*P*
Children’s sex role score ← Parents’ gender equality consciousness	0.402	0.117	0.094	4.271	0.000^*^	0.402	0.117	0.094	4.271	0.000^*^
Peer relationship ← Children’s sex role score	0.187	0.387	0.012	15.203	0.000^*^	0.187	0.387	0.012	15.203	0.000^*^
Parent–child relationship ← Children’s sex role score	0.092	0.383	0.009	10.415	0.000^*^	0.088	0.372	0.009	10.097	0.000^*^
Parent–child relationship ← Peer relationship	0.090	0.181	0.018	4.948	0.000^*^	0.095	0.194	0.018	5.304	0.000^*^
Parent–child relationship ← Parents’ gender equality consciousness	0.120	0.145	0.028	4.269	0.000^*^	0.036	0.045	0.028	1.311	0.190
Teacher-student relationship ← Parents’ gender equality consciousness	0.088	0.053	0.057	1.546	0.122	0.176	0.109	0.055	3.191	0.001^*^
Teacher-student relationship ← Children’s sex role score	0.043	0.090	0.019	2.256	0.024^*^	0.085	0.181	0.019	4.553	0.000^*^
Teacher-student relationship ← Peer relationship	0.222	0.224	0.037	5.986	0.000^*^	0.171	0.175	0.037	4.680	0.000^*^
Teacher-student relationship ← Parent–child relationship	0.635	0.320	0.079	8.079	0.000^*^	0.478	0.240	0.077	6.189	0.000^*^
Children’s gender equality consciousness ← Parents’ gender equality consciousness	0.543	0.533	0.034	16.116	0.000^*^	0.581	0.558	0.033	17.493	0.000^*^
Children’s gender equality consciousness ← Teacher-student relationship	0.052	0.084	0.015	3.555	0.000^*^	0.052	0.081	0.015	3.555	0.000^*^

^*^P < 0.05.

Table 8 shows the path differences of the structural equation models of children’s consciousness of gender equality among different school-aged groups. The differences were significant among different study period groups [ΔP(measurement covariances) = 0.00 < 0.05, ΔP(measurement covariances) = 0.00 < 0.05, ΔP(measurement residuals) = 0.00 < 0.05]. Therefore, there were study period differences in the model.

**Table 8:** Multi-group analysis across academic period (*n* = 1,312).

Pathway	Lower study period					Middle study period					Higher study period				
	Non-SC	SC	SE	C.R.	*P*	Non-SC	SC	SE	C.R.	*P*	Non-SC	SC	SE	C.R.	*P*
Children’s sex role score ← Parents’ gender equality consciousness	0.228	0.063	0.191	1.193	0.233	0.848	0.236	0.172	4.944	0.000^*^	0.436	0.127	0.147	2.969	0.003^*^
Peer relationship ← Children’s sex role score	0.215	0.401	0.026	8.248	0.000*	0.162	0.384	0.019	8.483	0.000^*^	0.167	0.343	0.020	8.481	0.000^*^
Parent–child relationship ← Children’s sex role score	0.092	0.368	0.012	7.827	0.000*	0.087	0.384	0.011	8.078	0.000^*^	0.087	0.357	0.010	8.666	0.000^*^
Parent–child relationship ← Peer relationship	0.139	0.298	0.022	6.352	0.000*	0.081	0.151	0.025	3.264	0.001^*^	0.060	0.120	0.021	2.934	0.003^*^
Parent–child relationship ← Parents’ gender equality consciousness	0.134	0.150	0.039	3.469	0.000*	0.060	0.074	0.036	1.670	0.095	0.120	0.143	0.033	3.686	0.000^*^
Teacher-student relationship ← Parents’ gender equality consciousness	0.198	0.111	0.075	2.626	0.009*	0.147	0.083	0.079	1.852	0.064	0.094	0.059	0.064	1.474	0.141
Teacher-student relationship ← Children’s sex role score	0.030	0.061	0.024	1.241	0.215	0.085	0.172	0.025	3.335	0.000^*^	0.073	0.158	0.021	3.498	0.000^*^
Teacher-student relationship ← Peer relationship	0.308	0.333	0.044	6.990	0.000*	0.162	0.138	0.056	2.909	0.004^*^	0.111	0.117	0.040	2.765	0.006^*^
Teacher-student relationship← Parent–child relationship	0.672	0.337	0.102	6.601	0.000*	0.574	0.262	0.108	5.303	0.000^*^	0.428	0.228	0.083	5.145	0.000^*^
Children’s gender equality consciousness ←Parents’ gender equality consciousness	0.409	0.362	0.056	7.333	0.000*	0.578	0.579	0.040	14.632	0.000^*^	0.560	0.568	0.034	16.710	0.000^*^
Children’s gender equality consciousness ← Teacher-student relationship	0.083	0.132	0.031	2.677	0.007*	0.061	0.109	0.022	2.764	0.006^*^	0.122	0.195	0.021	5.754	0.000^*^

^*^P < 0.05.

## 4 Discussion

### 4.1 Current status analysis of the consciousness of gender equality among school-aged children

The total score for the consciousness of gender equality of school-aged children was (17.46 ± 7.74), the total score for the consciousness of gender equality of girls was (18.19 ± 7.67), and the total score for the consciousness of gender equality of boys was (16.69 ± 7.75). Both male and female students had the lowest consciousness of gender equality in the field of occupation and relatively high consciousness of gender equality in the fields of family and school, consistent with the research results of Su et al. (2020). This indicates that school-aged children generally have serious occupational gender stereotypes. This may be related to China’s current occupational gender segregation. Yang and Zhang (2019) found that compared with men, it is more difficult for Chinese women to enter high-income industries. Therefore, school-aged children may be affected by the social environment of occupational gender segregation in China and may be likely to hold occupational gender stereotypes. Parents and teachers should not restrict children’s life and education but should encourage them to boldly choose various professions and help them establish an equal concept of gender in occupations to expand the possibility of children’s future career development.

The results of this study show that the consciousness of gender equality of children in all grades is on the rise, but it fluctuates slightly in the third and fourth grades. This is similar to the findings of Liao (2007), who showed that the third and fourth grades are a volatile and critical period for the development and change of children’s consciousness of gender equality. In the future, education should be carried out according to the characteristics of the consciousness of gender equality of children in different grades. Especially for third and fourth grade pupils, it is important to pay attention to their consciousness of gender equality and strengthen education on gender equality according to the psychological characteristics of children at this stage.

### 4.2 Structural equation model analysis of factors influencing the consciousness of gender equality among school-aged children

The results of this study showed that there was no significant difference in consciousness of gender equality between rural and urban children (*p* > 0.05). This finding is inconsistent with the results of Dong’s analysis (Dong, 2018) of Chinese women’s consciousness of gender equality based on the 2015 China Comprehensive Social Survey. This may be because this study was conducted only in Hunan Province, China, where the socio-cultural difference between rural and urban areas in the same region is small. However, Wang et al. (2021) found that with the development and popularization of the internet, rural residents’ gender concepts have gradually shown a trend toward equality and openness. The insignificant difference in consciousness of gender equality between rural and urban children in this study may also confirm the development and progress of the concept of gender equality in rural China and the promoting effect of the basic national policy of gender equality in China.

The results of the univariate analysis showed statistically significant differences in scores for consciousness of gender equality among school-aged children based on factors such as gender, age, grade, whether they were left-behind children (refers to children who remain in rural areas of China while one or both of their parents migrate to urban areas for work), parents’ emotional status, parents’ quarrels, number of same-sex friends, tendency to make friends, family monthly income, father’s educational level, mother’s educational level, child’s gender role, and parent’s gender role. Correlation analysis further revealed significant positive correlations between children’s consciousness of gender equality and their parents’ consciousness of gender equality, parents’ gender role total score, children’s gender role total score, parent–child relationship, and teacher-student relationship. In multiple regression analysis, only gender, age, parents’ consciousness of gender equality, teacher-student relationship, and parent–child relationship were included in the final equation, indicating that these variables are the most significant factors that influenced children’s consciousness of gender equality. Therefore, these variables were incorporated into the structural equation model for the final path analysis. Moreover, although children’s peer relationships did not directly correlate with consciousness of gender equality scores, they were significantly positively correlated with parent–child relationships, teacher-student relationships, and gender roles. Based on ecosystem theory and previous research (Bronfenbrenner and Ceci, 1994; Guo et al., 2014; Li and Lin, 2021; Zhao, 2021), we hypothesized that children’s peer relationships might indirectly affect their consciousness of gender equality through the mediating effects of parent–child and teacher-student relationships. The structural equation model results verified this hypothesis.

The results of the structural equation model showed that parents’ consciousness of gender equality, children’s gender roles, parent–child relationships, teacher-student relationships and peer relationships all had positive predictive effects on the consciousness of gender equality of school-aged children. Parents’ consciousness of gender equality had the strongest influence on school-aged children’s consciousness of gender equality. Gender roles, parent–child relationships, teacher-student relationships and peer relationships all play a part in mediating the influence of parents’ consciousness of gender equality on children’s consciousness of gender equality, similar to previous research results (Jacobs and Eccles, 1992; Lawson et al., 2015). Family is the first place for children’s socialization, and parents’ understanding of gender equality can not only provide a good example for children by helping them improve their consciousness of gender equality but also contribute to the improvement of parent–child relationships, teacher-student relationships and peer relationships as well as the cultivation of children’s androgynous gender roles. Therefore, in the process of education, in addition to the need to focus on educating male children and younger children, educators should integrate the concept of androgynous education, improve their own consciousness of gender equality, and create a good example for children. In the process of education, attention should also be paid to strengthening the close relationships among teachers, parents and children and promoting children’s recognition of their educational concepts to help children establish equal gender consciousness.

The results of the multi-group structural equation model showed that the structural equation model constructed in this study had no significant path differences between children of different genders but had significant path differences between children of different study periods. This indicates that although there is a significant difference in consciousness of gender equality between male and female children, there is no significant difference in the influencing factors and influencing paths of consciousness of gender equality. Study period differences not only lead to differences in children’s consciousness of gender equality but also affect the path relationship of children’s consciousness of gender equality. Compared with gender factors, children’s study period has a more important impact on the generation and development of their consciousness of gender equality. However, it may also be that because the subjects of this study were school-aged children, there was a small gap in the education levels and ages of children of different genders, which may have interfered with tests related to gender. Future studies can include people from more study periods for further testing. When formulating and implementing gender equality education programs for school-aged children, educators can ignore gender factors and implement the same gender equality education for boys and girls. However, these educational programs should be formulated according to the psychological characteristics of different children of different grades to effectively improve the consciousness of gender equality of school-aged children.

### 4.3 Recommendations and strategies for education on gender equality for school-aged children

Family and school are the primary environments for implementing gender equality education for school-aged children. Based on the analysis of the results, this study offers the following recommendations to enhance gender equality education for school-aged children.

In the family setting, parents should first address their own gender stereotypes and foster a household atmosphere that promotes gender equality, thereby enhancing their children’s consciousness of gender equality. Additionally, parents should strengthen the parent–child relationship, which is a crucial factor in enhancing children’s consciousness of gender equality. A positive relationship helps children align with their parents’ values and educational concepts (Zhang et al., 2019). Therefore, to improve children’s consciousness of gender equality, parents also need to respect their children, increase communication, and build a harmonious relationship to facilitate effective gender equality education within the family.

Schools play a vital role in implementing gender equality education. To ensure the smooth implementation of gender equality education, schools should first train a professional team of teachers to conduct gender equality teaching activities for school-aged children. Additionally, education should be tailored to the psychological characteristics of school-aged children by employing gentle methods and maintaining a positive teacher-student relationship to facilitate children’s absorption and understanding of gender equality knowledge. Furthermore, the results of this study indicate that androgynous gender roles enhance children’s consciousness of gender equality. Therefore, schools and teachers should incorporate gender role education into their curricula. This could include group games and physical exercises that require cooperation between boys and girls and helping children develop their own gender strengths while also learning the positive qualities of the opposite sex. These activities can promote peer relationships, foster androgynous gender roles, and cultivate a strong sense of gender equality.

## 5 Conclusion

Parents’ consciousness of gender equality, children’s gender roles, parent–child relationships and teacher-student relationships all have positive predictive effects on the consciousness of gender equality of school-aged children. Parents’ consciousness of gender equality has the strongest influence on school-aged children’s consciousness of gender equality. Gender roles, parent–child relationships, teacher-student relationships and peer relationships can all play a partially mediating role in the influence of parents’ consciousness of gender equality on that of children. The structural equation model constructed in this study is applicable to school-aged children of different genders, and there was a significant difference in the structural equation modeling of different school-aged children’s study period groups. This study provides theoretical support and reference for the subsequent formulation and implementation of gender equality education programs for school-aged children.

## 6 Limitations

First, this study included only 1,312 school-aged children in Hunan Province, China. The sample size was not large enough, and the sample was not representative. Future studies should consider expanding the study scope and sample size to include more school-aged children from different cultural backgrounds in the study at the macro system level. Second, due to the cross-sectional study method, the causal relationship between variables was based on theoretical analysis and a literature review, which require careful interpretation. Third, these data were collected during the COVID-19 pandemic, when some students’ school days may have been reduced under lockdown management. This may have weakened the influence of teachers and peers on children’s consciousness of gender equality. Finally, due to the potential mobility of teachers and classmates resulting from events such as school transitions or relocations, this study focused solely on the consciousness of gender equality of children and parents and did not measure the consciousness of gender equality of teachers and peers. It is hoped that future research will include perspectives from these additional groups to comprehensively explore the impact of family, school, and peer environments on school-aged children’s consciousness of gender equality.

## Data availability statement

The raw data supporting the conclusions of this article will be made available by the authors, without undue reservation.

## Ethics statement

The studies involving human/animal participants were reviewed and approved by Xiangya Nursing School of Central South University [No: E202165]. The studies were conducted in accordance with the local legislation and institutional requirements. Written informed consent for participation in this study was provided by the participants' legal guardians/next of kin.

## Author contributions

YL: Data curation, Investigation, Software, Writing – original draft, Writing – review & editing. JieZ: Writing – review & editing, Methodology. JL: Data curation, Writing – review & editing, Formal analysis. YC: Data curation, Formal analysis, Writing – review & editing. JinZ: Supervision, Writing – original draft, Writing – review & editing. MZ: Funding acquisition, Investigation, Software, Writing – original draft, Writing – review & editing.
